# Depletion of REF/Aly alters gene expression and reduces RNA polymerase II occupancy

**DOI:** 10.1093/nar/gku1278

**Published:** 2014-12-03

**Authors:** Sarah H. Stubbs, Nicholas K. Conrad

**Affiliations:** Department of Microbiology, University of Texas Southwestern Medical Center, 6000 Harry Hines Boulevard, Dallas, TX 75390-9048, USA

## Abstract

Pre-mRNA processing is mechanistically linked to transcription with RNA pol II serving as a platform to recruit RNA processing factors to nascent transcripts. The TREX complex member, REF/Aly, has been suggested to play roles in transcription and nuclear RNA stability in addition to its more broadly characterized role in mRNA export. We employed RNA-seq to identify a subset of transcripts with decreased expression in both nuclear and cytoplasmic fractions upon REF/Aly knockdown, which implies that REF/Aly affects their expression upstream of its role in mRNA export. Transcription inhibition experiments and metabolic labeling assays argue that REF/Aly does not affect stability of selected candidate transcripts. Instead, ChIP assays and nuclear run-on analysis reveal that REF/Aly depletion diminishes the transcription of these candidate genes. Furthermore, we determined that REF/Aly binds directly to candidate transcripts, supporting a direct effect of REF/Aly on candidate gene transcription. Taken together, our data suggest that the importance of REF/Aly is not limited to RNA export, but that REF/Aly is also critical for gene expression at the level of transcription. Our data are consistent with the model that REF/Aly is involved in linking splicing with transcription efficiency.

## INTRODUCTION

In the eukaryotic cell, a pre-mRNA must undergo multiple processing events to generate a mature mRNA. Many of these nuclear pre-mRNA processing steps including capping, splicing and 3′-end formation occur co-transcriptionally (1–4). In fact, pre-mRNA processing is not only temporally linked to RNA synthesis, but is also mechanistically linked. That is, processing does not simply occur co-transcriptionally, but the transcription and processing machineries interact in a fashion that renders RNA processing more efficient when coupled with transcription (4,5). RNA polymerase II (pol II) is uniquely suited to facilitate co-transcriptional pre-mRNA processing largely through its repetitive carboxyl-terminal domain (CTD) that recruits various RNA processing factors throughout the transcription cycle (6). Reversible phosphorylation of multiple residues of the CTD facilitates the recruitment and activities of RNA processing factors (7,8). As a result, truncation of the CTD results in severe defects in 3′-end processing, splicing (9,10) and cell viability (11–13), thereby demonstrating the importance of coupling between transcription and RNA processing.

Much work has been done demonstrating that cells link transcription with downstream events in RNA processing, but recent investigations suggest that RNA processing can, in turn, modulate transcription rates. For example, several aspects of pre-mRNA splicing have been associated with transcription. Splicing efficiency and splice site mutations have been shown to impair transcription activity by decreasing assembly of the pre-initiation complex (PIC) (14) and repositioning the active transcription marker, H3K36me3 (15). Furthermore, first exon length is an important determinant of the active chromatin signatures H3K4me3 and H3K9ac, as well as transcription factor density (16). Not only are splicing elements within the gene important for determining transcription activity, but splicing proteins are also linked to transcription activity. Depletion of the splicing factor SC35 causes accumulation of pol II in the gene body and reduces elongation efficiency (17). SC35 associates with the 7SK complex at gene promoters and facilitates release of P-TEFb from the 7SK complex to enable transcription elongation (18). Furthermore, the spliceosomal U snRNPs as well as splicing signals in the nascent transcript stimulate transcription elongation (19). Additionally, in yeast the Prp19 complex was found to have a role in transcription elongation by stabilizing recruitment of TREX to RNA pol II (20). Other steps in pre-mRNA processing aside from splicing have also been linked to transcription activity. The cap-binding complex (CBC) interacts with P-TEFb (Cdk9 and Cyclin T1) and affects Ser-2 phosphorylation (21). In yeast, deletion of the CBC results in decreased recruitment of the Bur and Ctk complexes, causing lower Ser-2 phosphorylation and H3K36 methylation (22). Disruption of 3′-end processing results in decreased TFIIB and TFIID at promoters and causes reduced transcription (23). These data provide evidence that cross-talk between gene expression events is bidirectional, and suggest an added layer of complexity between transcription and mRNA processing. However, little is known about the mechanisms and factors involved.

Pre-mRNA splicing changes ribonucleoprotein (RNP) composition to facilitate downstream events in gene expression. Subsequent to intron removal by the spliceosome, the exon junction complex (EJC), is deposited ∼20 nucleotides (nt) upstream of the exon–exon junction (24). The EJC and the CBC promote recruitment of the TREX (transcription-export) complex to the 5′-most exon (25–29). The TREX complex is a highly conserved multi-protein complex composed of REF/Aly, UAP56, CIP29 and the THO complex (Hpr1, TEX1, Thoc2, Thoc5, Thoc6, Thoc7). Recently, several additional TREX complex members were identified that appear to be unique to the mammalian TREX complex, including ZC11A, PDIP3 and Chtop (30,31). Deposition of TREX results in recruitment of TAP/NXF1 to the mRNA where it binds REF/Aly, displacing UAP56 and triggering the transfer of the mRNA from REF/Aly to TAP/NXF1 (32,33). TAP/NXF1 and its partner p15 interact with the nuclear pore to assist in export of the mature transcript to the cytoplasm (34,35).

One TREX component, REF/Aly, has been implicated in multiple processes including RNA export, nuclear RNA stability and transcription. REF/Aly's importance is underscored by the fact that it is conserved from yeast to humans (27,36), but depletion of REF/Aly homologs results in a variety of phenotypes. Export of bulk mRNA is not significantly affected when REF/Aly is knocked down in *Caenorhabditis elegans* and *Drosophila melanogaster* (37,38). However, deletion of the REF/Aly homolog in yeast, Yra1, prevents bulk mRNA export (27,36). Depletion of REF/Aly in human cells also displays a variety of phenotypes. Some reports show a strong nuclear accumulation of poly(A) RNA upon REF/Aly depletion, while other studies suggest a more modest retention of poly(A) RNA in the nucleus (39–42). Although export phenotypes vary, REF/Aly is essential for cell viability in both *Drosophila* and humans. Differences in phenotype may be explained by the presence of redundant export factors (43), up-regulation of other export factors (40) or experimental procedures. Nevertheless, these data suggest that REF/Aly functions in the export of at least some specific mRNAs.

Prior to its definition as an mRNA export factor, REF/Aly was implicated in transcriptional control. REF/Aly interacts with AML-1 and LEF-1, two transcription factors that are involved in activation of the T cell receptor α gene (TCRα) enhancer (44). REF/Aly increases the binding of AML-1 and LEF-1 to DNA, and overexpression led to an increase in the activation of the TCRα enhancer complex. Additionally, REF/Aly exhibits chaperone functions by promoting dimerization of basic region-leucine zipper (bZIP) transcription factors which increases the association of bZIP proteins to DNA (45). REF/Aly also associates with Iws1, a protein that interacts with the elongation factor, Spt6 (46). Furthermore, Dominguez-Sanchez *et al.* demonstrated that depletion of REF/Aly as well as other THO/TREX components results in transcription elongation defects and that THO/TREX members are involved in genome stability and prevention of R-loop formation (47). This is consistent with previous data linking yeast transcription and genome integrity (48). Thus, REF/Aly may provide additional functions in RNA synthesis besides its function in RNA export, and perhaps links REF/Aly with transcription elongation.

REF/Aly is also known to stabilize at least one RNA, PAN RNA, a Kaposi's sarcoma-associated herpesvirus nuclear non-coding RNA (49). Artificial tethering of REF/Aly to PAN RNA is sufficient to increase the half-life of PAN RNA. However, the transcript remains in the nucleus, demonstrating that the REF/Aly export function is not required for the stabilization activity. In addition, tethering of REF/Aly increases the nuclear abundance of an inefficiently spliced intron-containing reporter (40). Furthermore, the TREX complex appears to increase the nuclear stability of endogenous intronless mRNAs (50,51). These data suggest that TREX components, particularly, REF/Aly, are capable of stabilizing nuclear transcripts, independent of their roles in RNA export.

Here, we aimed to identify endogenous RNAs affected by REF/Aly early in their biogenesis. In particular, we looked for transcripts that were destabilized or had decreased transcription in the absence of REF/Aly. Using an RNA-Seq approach, we identified transcripts whose levels were altered upon REF/Aly depletion and focused on several candidates that were down-regulated in the absence of REF/Aly in both the nuclear and cytoplasmic fractions, suggesting that the changes in expression were not driven solely by mRNA export defects. Half-life determinations, metabolic labeling assays, pol II chromatin immunoprecipitation (ChIP) and nuclear run-on (NRO) assays showed that REF/Aly enhances the transcription of specific target genes. Additionally, we show that REF/Aly binds directly to candidate transcripts, consistent with a direct effect of REF/Aly on the candidates. Our data suggest that the importance of REF/Aly is not limited to RNA export, but that REF/Aly is also critical for gene expression at the level of transcription and may be involved in linking transcription efficiency with splicing.

## MATERIALS AND METHODS

### Cell culture and transfection

293A-TOA cells were grown in Dulbecco's modified Eagle's medium (Sigma) containing 10% tetracycline-free fetal bovine serum (Clontech), 1× penicillin-streptomycin (Sigma), 2 mM L-glutamate and 100 μg/ml G418 (Fisher BioReagents).

293A-TOA cells were transfected with a final concentration of 16 nM siRNAs using Lipofectamine RNAiMax (Invitrogen) according to manufacturer instructions. For siREF samples, cells were transfected with a pool of two (8 nM each) *Silencer* Select siRNAs targeting REF (Ambion). Control samples were transfected with *Silencer* Select Negative Control #2 siRNA. Cells were transfected with siRNAs for 24 h before replating on larger plates. For example, for RNA-Seq and ChIP sample preparation, 293A-TOA cells were initially plated on 6-well plates for siRNA transfection. Twenty-four hours after transfection, cells were trypsinized, re-plated on 10-cm plates and allowed to grow for 72 more hours, 96 h total. We found that efficient knockdown required cell division, not just additional time, presumably because the REF/Aly protein is stable. Ultraviolet (UV) crosslinking experiments were performed in 10-cm plates with 10 μg Flag-REF2–I plasmid DNA (52) using Transit293 (Mirus) according to manufacturer instructions.

### Antibodies and immunoblotting

All antibodies were purchased commercially from the following sources: REF (Sigma, clone 11G5), Pol II (Covance, clone 8WG16), H3K36me3 (Abcam, ab9050), hnRNP C1/C2 (clone 4F4), TAP (Proteintech, 10328–1-AP), Hpr1 (Abcam, ab487), UIF (Bethyl Laboratories, a303–525a), UAP56/URH49 (Santa Cruz Biotechnology, sc271395). Protein was harvested following REF/Aly knockdown with siRNAs at various time points, separated on sodium dodecyl sulphate (SDS)-polyacrylamide gel electrophoresis, and quantitative western blots were performed using infrared detection with an Odyssey Fc machine. Quantification of western blots was performed using *Image Studio* software (LI-COR Biosciences).

### Nucleocytoplasmic fractionation

For RNA-Seq sample preparation, 293A-TOA cells were transfected with 16 nM of siREF (pooled) or siControl and grown on three 10-cm plates per siRNA for 96 h. After knockdown, cells were harvested from one 10-cm plate, washed with ice-cold phosphate-buffered saline (PBS), and resuspended in 500 μl Buffer I (0.32 M Sucrose, 3 mM CaCl_2_, 2 mM MgCl_2_, 0.1 mM ethylenediaminetetraacetic acid (EDTA), 10 mM Tris pH 8.0) which was immediately added to 5 ml Tri-Reagent (Molecular Research Center); this is the ‘total’ fraction. Cells were then harvested and combined from the other two 10-cm plates and resuspended in 1 ml Buffer I with fresh 1 mM DTT, 0.04 U/μl RNaseIN and 0.5% TritonX-100, and incubated on ice for 5 min. Samples were then centrifuged at 500 × *g* for 5 min at 4°C. To obtain the ‘cytoplasmic’ fraction, the supernatant was removed and added to 10 ml Tri-Reagent. The pellet was then resuspended in 1 ml Buffer I and added to 10 ml Tri-Reagent; this is the ‘nuclear’ fraction.

### RNA-Seq

Fractionated RNAs were selected two times using the Poly(A) Purist Kit (Ambion) to enrich for polyadenylated RNA following manufacturer specifications. Analysis using an Agilent Bioanalyzer indicated that rRNA contamination in samples was minimal. Two biological replicates were prepared and sequenced using the SOLiD 5500 sequencing platform. Reads were mapped back to the UCSC hg19 reference genome using LifeScope. Mapped reads were then post-processed for transcript assembly and these reads were assembled into their respective transcripts using Cufflinks version 1.2.0 (53). Transcript abundances were estimated using their FPKM (fragments per kilobase of exon per million reads mapped) after upper-quartile normalization. These assembled transcripts and abundance estimations were then passed to Cuffdiff for differential expression analysis between conditions. Differentially expressed genes (DEGs) were defined as those comparisons whose false discovery rate (FDR)-adjusted *P*-value was <0.05, as determined by Benjamini–Hochsberg correction. Because transcripts with very low expression can be unreliable, we omitted the genes with the lowest 20% of expression levels.

### RNA procedures

All RNA analyzed by reverse transcriptase-quantitative polymerase chain reaction (RT-qPCR) was treated with RQ1 DNase (Promega) before the reverse transcription (RT) reaction. RT reactions were performed using SuperScript™ II RT (Invitrogen) or M-MuLV-RT (New England Biolabs) and random hexamers (Sigma) or dT_20_ (fractionation experiments) as per the manufacturer's protocol. cDNAs were then treated with RNase A and RNase H for 30 min at 37°C and purified with phenol:chloroform:isoamyl alcohol (PCA). cDNAs were resuspended in 20 μl H_2_O and diluted in an assay-dependent manner. Primer sequences and efficiencies are described in Supplementary Table S3. Relative quantities (RQ) were determined based on amplification efficiency and Ct value. To determine the relative expression of candidate transcripts after fractionation, single knockdown or knockdown over time, RQ values were normalized to RQ values of 7SK RNA.

To assess relative stability of candidate transcripts, cells transfected with REF or Control siRNAs were treated with 1 ug/ml Actinomycin D to inhibit transcription. Cells were harvested at various time points from 0 to 4 h by addition of Tri-Reagent. RNA was extracted and analyzed by RT-qPCR to determine RNA abundance after transcription shut-off. Transcription shut-off RT-qPCR was performed using a one-step reaction with MultiScribe Reverse Transcriptase (Invitrogen).

### ChIP

293A-TOA cells were grown on 6-well plates and transfected with 16 nM siControl or pooled siREF. Twenty-four hours after transfection, cells were replated on 10-cm plates and allowed to grow until REF/Aly was depleted for 96 h. To crosslink cells, 0.75% formaldehyde was added to each plate and plates were rocked for 10 min at room temperature. Formaldehyde was quenched by addition of 125 mM glycine. Cells were harvested from plates, washed three times with PBS and resuspended in 500 μl RIPA buffer (1% NP-40, 0.5% sodium deoxycholate, 0.1% SDS, 150 mM sodium chloride, 50 mM Tris-HCl [pH 8.0], 2 mM EDTA) containing protease inhibitors. Cells were sonicated for 45 cycles (30 s on and 30 s off per cycle) at 4°C using a Digenode Bioruptor on the high setting to shear DNA to ∼200–300 base pairs. Sheared chromatin was centrifuged at max speed for 10 min at 4°C and then DNA was measured using a NanoDrop Spectrophotometer. Samples were normalized and ∼200 μg DNA was added to protein A beads conjugated with either 8WG16 (4 μl) or H3K36me3 (3 μg) antibodies. DNA was immunoprecipitated for at least 2 h at 4°C, and then washed with 1 ml of the following washes: (i) RIPA, (ii) low salt wash (0.1 SDS, 1% TritonX100, 2 mM EDTA, 20 mM Tris (pH 8.0), 150 mM NaCl), (iii) high salt wash (0.1 SDS, 1% TritonX100, 2 mM EDTA, 20 mM Tris (pH 8.0), 500 mM NaCl), (iv) LiCl wash (0.25 M LiCl, 1% NP40, 1% NaDeoxycholate, 1 mM EDTA, 10 mM Tris pH 8.0), (v) T.E. buffer (10 mM Tris pH 8.0, 1 mM EDTA), (vi) T.E. buffer. Chromatin was eluted by addition of 200 μl elution buffer (100 mM NaHCO_3_, 1% SDS) and incubation at 65°C for 15 min. To reverse protein crosslinks and degrade protein, chromatin was incubated at 65°C overnight in elution buffer containing 20 mg/ml Proteinase K and 40 μl Tris pH 6.8. Chromatin was purified using PCA (25:24:1) and ethanol precipitation and DNA was analyzed by qPCR. ChIP amplicon primer sequences and efficiencies can be found in Supplementary Table S3.

### UV crosslinking

UV crosslinking experiments were performed in 293A-TOA cells as previously described (49,54) and immunoprecipitated RNA was analyzed by RT-qPCR. For UV crosslinking experiments, RQ values of −reverse transcriptase samples were subtracted from +reverse transcriptase RQ values, and immunoprecipitation efficiency was determined by calculating the pellet-to-input ratio. All samples were expressed relative to signal from ACTB to control for experimental variation.

### 4-thiouridine labeling and click-IT nascent RNA capture

For 4-thiouridine (4SU) labeling experiments (55,56), 293A-TOA cells were pulsed for 2 h with 2.5 μM 4SU. Cells were then harvested with Tri-Reagent and collected RNA was treated with RQ1 DNase. Note that 15 μg RNA was biotinylated by addition of buffer containing 10 mM Tris pH 7.5, 1 mM EDTA, 0.2 mg/ml Biotin-HPDP (Pierce) in a final volume of 125 μl. The reaction was incubated at room temperature for 3 h in the dark. Next, the sample volume was brought to 250 μl in 1 M ammonium acetate and extracted with 250 μl Chloroform:isoamyl-OH (24:1). The aqueous phase was added to 700 μl 100% ethanol and precipitated overnight at −20°C. Dynal MyOne Streptavidin T1 beads (10 μl per sample) were washed three times in MPG-I (1M NaCl, 10 mM EDTA, 100 mM TrisHCl pH 7.5, 0.1% Igepal) and resuspended in 170 μl MPG-I. Precipitated RNA from the biotinylation reaction was washed with 70% ethanol, resuspended in 30 μl H_2_O and heated at 65°C for 5 min. RNA was added to the washed Dynal Streptavidin T1 beads for a final volume of 200 μl and incubated at room temperature for 30 min with nutation. Beads were washed eight times using the following washes: (i) MPG-I, (ii) 65°C MPG (1 M NaCl, 10 mM EDTA, 100 mM TrisHCl pH 7.5), (iii–vi) MPG-I, (vii) MPG without NaCl, (viii) MPG-1:10 (MPG-I diluted 1:10). Selected RNAs were eluted by first adding 200 μl elution buffer (MPG 1:10 + 5% β-mercaptoethanol) and incubated at room temperature for 5 min. Eluted RNA was moved to a new tube and beads were resuspended in another 200 μl elution buffer and incubated at 65°C for 10 min. Eluted fractions were combined and RNA was PCA extracted and ethanol precipitated. Relative expression of RNAs was measured by RT-qPCR.

Analysis of bulk poly(A) RNA with ethynyl uridine (EU) was performed as previously described (57) using 1.5 μg RNA. Selected RNA was eluted from beads by addition of RNase T1 after the final wash. Note that 30 μl of RNA/beads were incubated with 20 mM Tris pH 6.8, 8 U RNaseIN and 1000 U/ul RNase T1 for 15 min at 37°C. The reaction was stopped by addition of 170 μl G-50 Buffer (20 mM Tris pH 7.5, 0.25% SDS, 0.3 M sodium acetate, 2 mM EDTA) containing 0.1 mg/ml Proteinase K and 1.5 μl glycoblue. RNA was extracted with an equal volume of PCA and ethanol precipitated. The entire sample was then assayed by northern blot with a 1.8% agarose gel and a 5′-end labeled dT_40_ probe.

### NRO assays

We developed a NRO assay using 4-thiouridine 5′-triphosphate (4SUTP; TriLink Biotechnologies) based on two established protocols (58,59). Approximately 2 × 10^7^ cells (siControl or siREF as indicated) were used per sample. Cells were harvested by trypsin digestion, trypsin was quenched in ice-cold media and cells were washed in ice-cold 1× PBS. Cell pellets were then incubated for 5 min on ice in 1 ml of HLB (10 mM Tris-HCl (pH 7.5), 10 mM NaCl, 2.5 mM MgCl_2_) supplemented with 1 mM DTT and 0.5% IGEPAL CA-630. The solution was then underlaid with 1 ml of HLB supplemented with 25% sucrose (w/v), 1 mM DTT and 0.5% IGEPAL CA-630 to create a step gradient and then centrifuged at 600 × *g* for 5 min at 4°C. After discarding the supernatant, the pellets were resuspended in 60 μl of 2× TXN buffer (20 mM TrisHCl (pH8.0), 180 mM KCl, 10 mM MgCl_2_, 50% Glycerol, 5 mM DTT, 40 U RNasin). Nts were then added to final concentrations of 250 μM ATP, GTP and CTP and 1 μM 4SUTP. The ‘-4SU’ samples substituted UTP for 4SUTP. Sarkosyl was added to 0.5% and transcription was allowed to proceed for 5 min at 30°C prior to stopping the reaction by addition of 4 ml Tri-Reagent. RNA was extracted and treated with DNase. Next, 40 μg of RNA was subjected to limited hydrolysis on ice in a volume of 50 μl of 200 mM NaOH for 4 min. To neutralize the solution, a mix of 50 μl 1M TrisHCl (pH 6.8), 10 μl 3M NaOAc (pH 5.2) and 1 μl glycoblue was added and samples were ethanol precipitated.

Due to the lower signal-to-noise for NROs, modifications to the 4SU-biotinylation and SA selection protocols were necessary. To biotinlylate RNAs, the hydrolyzed RNA samples were resuspended in 50 μl H_2_O, heated to 65°C for 3 min, cooled on ice for 1 min and 50 μl of a 4× biotinylation buffer was added (80 mM NaOAc, pH 5.2, 4 mM EDTA, 0.4% SDS). Samples were warmed to 25°C prior to adding 100 μl of 0.4 mg/ml biotin-HPDP (in dimethylformamide) for a final concentration of 0.2 mg/ml biotin-HPDP. After a 3-h incubation at 25°C, samples were chloroform extracted once, then ammonium acetate was added to 1 M, 1 μl of glycoblue was added and the aqueous volume brought to 200 μl with H_2_O. After two more chloroform extractions, the samples were ethanol precipitated. For SA selection, precipitated RNA was washed with 70% ethanol, resuspended in 60 μl H_2_O and heated at 65°C for 5 min. For each sample, 20 μl of Dynal MyOne Streptavidin T1 bead slurry was washed three times in MPG-1:10 and pre-blocked for 1 h in 1 ml of MPG-1:10 supplemented with 0.1% SDS, 0.1 mg/ml sheared salmon sperm DNA, 0.1 mg/ml poly(A) RNA (Sigma) and 0.1 mg/ml Torula yeast RNA (Sigma). This pre-blocked solution (950 μl) was then added to the heated RNA samples and binding proceeded for 1 h at room temperature. Beads were washed 11 times using the following washes: (i) MPG-1:10, (ii and iii) 55°C MPG-1:10 (without IGEPAL), (iv) MPG-1:10, (v–vii) MPG-I, (viii) MPG-1:10, (ix and x) MPG-I, no NaCl, (xi) MPG-1:10. Selected RNAs were eluted by two successive steps of 200 μl elution buffer (MPG 1:10 + 5% β-mercaptoethanol) incubated at room temperature for 5 min. Eluted fractions were combined and RNA was PCA extracted, chloroform extracted and ethanol precipitated. Relative expression of RNAs was measured by RT-qPCR.

## RESULTS

### Depletion of REF/Aly alters RNA expression

Our goal was to find specific mRNAs that were affected by REF/Aly early in their biogenesis, particularly at the levels of nuclear RNA stability or transcription. Therefore, we wanted to exclude RNAs that were affected by REF/Aly primarily at the level of mRNA export. To identify candidate transcripts, we employed RNA-Seq to analyze RNAs in total, nuclear or cytoplasmic fractions upon siRNA-mediated REF/Aly knockdown. In the simplest scenario, RNAs that are exported in a REF/Aly-dependent fashion will decrease in the cytoplasm and increase in the nucleus upon REF/Aly depletion, but be relatively unchanged in the total. However, the factors involved in nuclear and cytoplasmic RNA decay differ, and this simple model does not account for inherent differences in RNA decay rates between the nuclear and cytoplasmic compartments. In principle, if REF/Aly is necessary for the export of a transcript, its total levels may decrease upon REF/Aly knockdown if the transcript is more rapidly degraded when sequestered in the nucleus. However, if REF/Aly acts exclusively at the level of mRNA export, the nuclear levels should not decrease upon REF/Aly knockdown. As such, we reasoned that if a transcript displays decreased levels in both nuclear and cytoplasmic compartments upon REF/Aly depletion, then either the nuclear stability or transcription of that mRNA must be altered. Therefore, in our RNA-Seq experiments we examined the effects of REF/Aly depletion on unfractionated cells, as well as nuclear and cytoplasmic fractions, and focused on transcripts reproducibly down-regulated in all three samples. Following identification of transcripts that are down-regulated in the nucleus, cytoplasm and total samples, we can subsequently determine whether REF/Aly acts on their stability or transcription.

**Figure 1.**
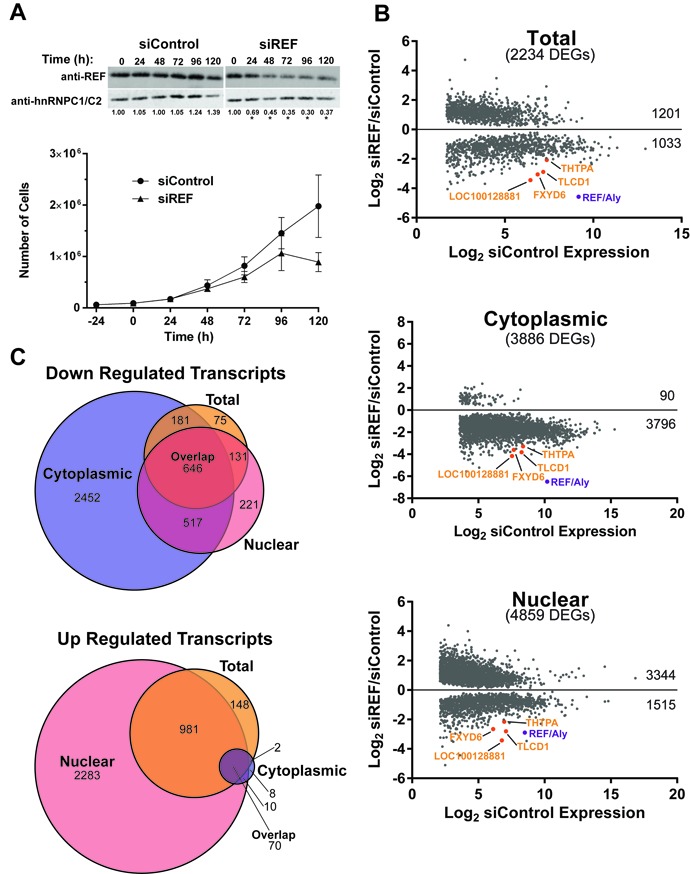
REF/Aly depletion alters RNA expression profiles. (A) Top: Representative western blots of REF/Aly depletion over time. Quantitative western blots were performed on whole cell extracts from cells depleted with either REF/Aly or control siRNAs for the indicated times. Proteins were detected with REF/Aly antibodies and hnRNP C1/C2 as a control. Numbers below blots indicate relative REF/Aly protein levels, *P < 0.01. Bottom: Growth curves of cells treated with REF or control siRNAs. Time 0 indicates the day of siRNA transfection. Error bars are standard deviation from the mean (*n* = 3). (B) Scatter plots showing only the DEGs from each of the three samples as indicated. The *y*-axis is the differential of RNA levels between REF/Aly and control siRNAs, while the *x*-axis is the expression levels of the control siRNA sample. Numbers on the right side of the graphs indicate number of DEGs with increased expression (above *x* = 0) and decreased expression (below *x* = 0). Note that the genes with the lowest 20% expression were omitted. (C) Venn diagram comparing the overlap of transcripts down-regulated (top) or up-regulated (bottom) upon REF/Aly depletion in the cytoplasmic (blue), total (orange) and nuclear (pink) samples. Numbers indicate number of DEGs identified in each sample. ‘Overlap’ indicates the number of genes that were found to be either down-regulated or up-regulated in all three samples.

To determine the proper conditions for siRNA-mediated knockdown of REF/Aly, 293A-TOA cells (60) were transfected with siRNAs targeting REF/Aly or a control siRNA. Cells were counted and protein was harvested every 24 h for 5 days (120 h) to monitor changes in cell growth and to determine the length of time required to deplete REF/Aly protein levels. REF/Aly protein levels diminished gradually over time and by 96 h 70% of the protein was depleted (Figure 1A). Cell growth began to slow at 72 h but cells did not stop doubling until after 96 h, consistent with previously published observations (40). Gross cell morphology, as assessed by light microscopy, was not appreciably affected by REF/Aly depletion until after 96 h (data not shown). Similar to results observed in *Drosophila* cells (37), depletion of REF/Aly in 293A-TOA cells did not result in a complete accumulation of poly(A)+ transcripts in the nucleus, but increased nuclear signal was nonetheless observed (Supplementary Figure S1). To maximize REF/Aly depletion and minimize changes in cell growth, we chose to perform RNA-Seq experiments following a 96-h depletion of REF/Aly, however, confirmatory analysis at earlier time points was also performed (Figure 5). Additionally, we examined TREX component levels following a 96-h depletion of REF/Aly to ensure that changes in RNA levels were not the result of disruption or degradation of the TREX complex following REF/Aly knockdown (Supplementary Figure S2). Consistent with previously reported data, UIF protein levels increased upon REF/Aly knockdown (40), while Hpr1 and TAP levels remained the same.

**Figure 2.**
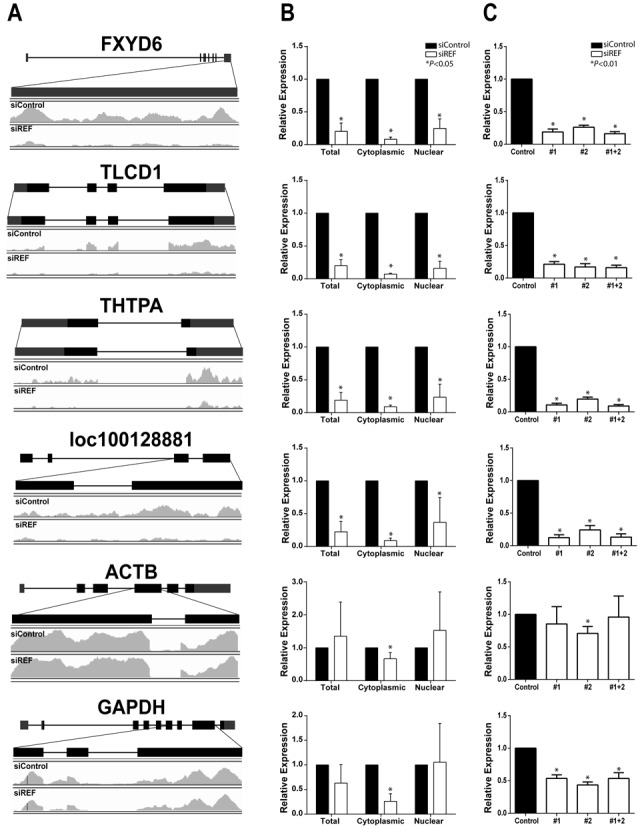
Validation of RNA-Seq candidates. (A) Diagram of candidate genes identified from RNA-Seq analysis. Mapped reads from the total fraction are shown for samples treated with siControl (top) or siREF (bottom) for area of gene indicated above traces. (B) RT-qPCR validation of candidate expression level following REF/Aly depletion and fractionation into nuclear and cytoplasmic fractions. (C) Relative expression in the total fraction of candidate transcripts measured by RT-qPCR following REF/Aly depletion with individual siRNAs (siREF #1 or siREF #2) or pooled siRNAs (siREF #1+2). Each row in (A), (B) and (C) all represent the same gene (for example, the first row represents FXYD6). RQ values in (B) and (C) were normalized to 7SK RNA expression; error bars are standard deviation from the mean (*n* = 3).

**Figure 3.**
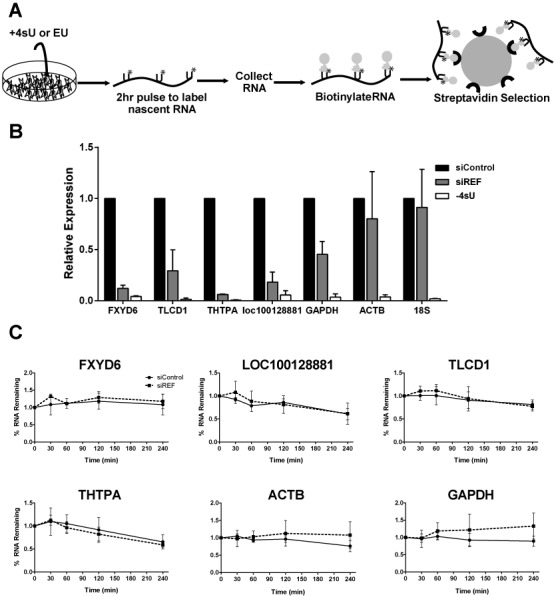
Decreases in candidate RNA levels occur early in mRNA biogenesis and are not due to decreased stability. (A) Schematic of uridine analog RNA labeling experiments; see text for details. (B) Analysis of newly made transcript levels by RT-qPCR in a 4SU labeling experiment. The ‘-4SU’ samples were transfected with siControl but no 4SU was added during the pulse. (C) Decay curves of candidate transcripts following transcription inhibition with 1 μg/ml ActD. Dashed lines represent cells treated with siREF and solid lines represent cells treated with siControl. Experiments in (B) and (C) were performed three times and error bars represent standard deviation from the mean.

**Figure 4.**
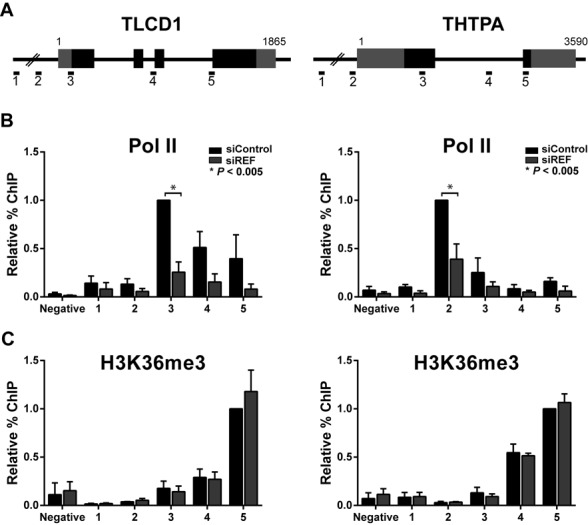
REF/Aly knockdown results in decreased pol II occupancy on candidate genes. (A) Diagram of TLCD1 and THTPA loci and amplicons used for ChIP experiments. (B) Relative ChIP signals for pol II are shown in the presence (black bars) or absence (gray bars) of REF/Aly. ChIP signal is normalized to the siControl signal at the amplicon closest to the TSS (TLCD1 #3, THTPA #2). The negative amplicon is located on chromosome 17 in a region that is at least 20 kb away from an annotated gene. (C) Relative ChIP signals for H3K36me3 in the presence or absence of REF/Aly as in part (B), except that ChIP signal is normalized to the siControl signal at the 3′-most amplicon (TLCD1 #5, THTPA #5). Error bars represent standard deviation from the mean (*n* = 3). *P-*values were calculated using two-tailed, unpaired Student's *t*-test.

**Figure 5.**
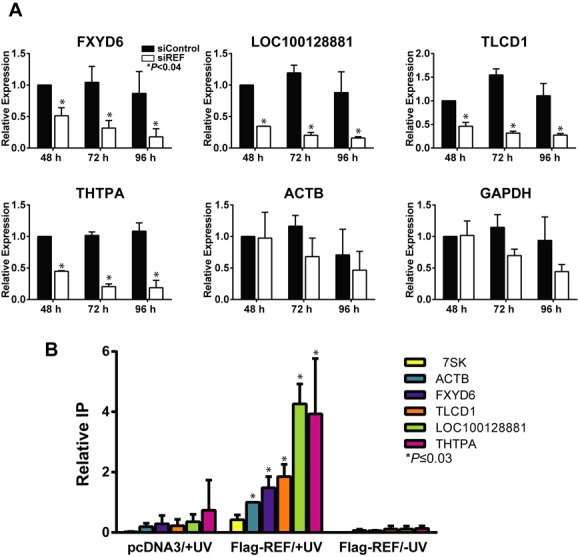
Candidate RNAs levels decrease quickly upon REF/Aly depletion and bind REF/Aly. (A) Relative expression of candidate transcripts were assayed by RT-qPCR analysis after a 48-, 72- or 96-h knockdown of REF/Aly. Expression is represented relative to siControl sample after a 48-h knockdown. Error bars represent standard deviation from the mean (*n* = 3). (B) Results from UV crosslinking RT-qPCR experiments. Cells were transfected with Fl-REF or a vector (pcDNA3) control and treated with UV as indicated. The bars represent the mean percent immunoprecipitation of RNA relative to ACTB, and error bars represent standard deviation from the mean (*n* = 3). We performed two different two-tailed, unpaired Student's *t*-tests on our data. First, we compared the +UV/Fl-REF samples to the −UV/Fl-REF samples from the same transcript. In each case, these values were statistically significant (*P* < 0.03; data not shown). Second, we compared each +UV/Fl-REF sample with the 7SK +UV/Fl-REF control, which is not expected to bind to REF/Aly (asterisks).

To prepare samples for RNA-Seq, cells were treated for 96 h with siRNAs targeting REF/Aly or with a control siRNA. Polyadenylated RNA was collected from unfractionated cells (total), nuclear or cytoplasmic fractions. The purity of the nuclear and cytoplasmic fractionation was verified by examination of nuclear pre-mRNAs and their cytoplasmic mRNA counterparts (Supplementary Figure S3). Two biological replicates were sequenced, mapped and DEGs were identified (53) (see Materials and Methods). After removing the lowest expressing genes (bottom 20%), we identified 2234 genes that were differentially expressed in the total sample, 3886 in the cytoplasmic fraction and 4859 in the nuclear fraction (Figure 1B). As expected, UAP56 (DDX39A) was one of the 70 genes up-regulated in all three samples. In support of REF/Aly's function in RNA export, approximately two-thirds of the nuclear DEGs were up-regulated, and the vast majority (98%) of DEGs in the cytoplasmic fraction were down-regulated. Detailed workflow, fold-changes, statistics and gene names for all samples are given in Supplementary Table S1.

As described above, our goal was to identify transcripts whose expression is enhanced by REF/Aly upstream of export, so we focused on transcripts that were down-regulated in all samples upon REF/Aly depletion and found 646 DEGs (Figure 1C, top, overlap). Gene ontology analysis (61,62) did not identify any enriched terms for candidates identified in the RNA-Seq analysis. Four candidate RNAs, FXYD6, TLCD1, THTPA and loc100128881, were chosen for further analysis because they were decreased greater than 4-fold upon REF/Aly depletion (Figure 1B, orange).

### Validation of RNA-Seq candidates

As expected, visual inspection of the mapped reads showed larger peaks in the siControl sample compared to the siREF/Aly sample, corresponding with the identified DEGs (Figure 2A). To confirm RNA-Seq results, REF/Aly was knocked down in 293A-TOA cells for 96 h, cells were fractionated and RNA was analyzed by RT-qPCR. In accordance with RNA-Seq results, expression of candidate transcripts was decreased in the total, cytoplasmic and nuclear fractions when REF/Aly was depleted (Figure 2B). Our RNA-Seq analysis used a two-siRNA pool targeting REF/Aly mRNA. To ensure that changes in expression levels were not a result of off-target effects from either of the siRNAs, each transcript was assayed for expression by RT-qPCR following depletion of REF/Aly with a single siRNA. All four candidate transcripts showed decreased RNA levels with single siRNA knockdown (Figure 2C). Importantly, decreased expression closely resembled knockdown with the siRNA pool, strongly supporting the conclusion that the candidate RNA levels are decreased as a result of REF/Aly depletion and are not due to off-target effects of either siRNA. Interestingly, we observed apparent decreases in both ACTB and GAPDH levels even though they were not identified as DEGs. We suspect this is due to the smaller fold-changes in these mRNAs coupled with higher variability in the responses. Both the magnitude and reproducibility of changes in expression constitute important parameters defining the statistical significance cutoffs for the identification of DEGs (53). We conclude that the RNA-Seq analysis uncovered transcripts whose abundance is dictated by REF/Aly, but that other transcripts may also be affected. In addition, because the transcripts decrease in the cytoplasm and the nucleus, our data suggest that the diminished expression is not solely due to inefficient mRNA export.

### Changes in RNA levels are not due to a decrease in RNA stability

As described above, we hypothesized that REF/Aly acts early in biogenesis of our selected target mRNAs. However, examination of steady-state RNA levels can be misleading because some transcripts might have been generated prior to functional knockdown of REF/Aly. In order to look at changes in RNA accumulation over a brief window subsequent to REF/Aly knockdown, we metabolically labeled RNAs using the modified uridine analog, 4-thiouridine (4SU), to capture newly made RNAs (55,56). Upon addition to the media, 4SU is taken up by cells and incorporated into nascent transcripts (Figure 3A). The analog-containing RNA can then be biotinylated using a thiol-disulfide exchange reaction with HPDP-biotin, captured on streptavidin beads and detected by RT-qPCR. This assay allows for RNAs made during the 2-h pulse to be isolated from RNAs made before the pulse. By performing the pulse after REF/Aly knockdown, we can ensure that the transcripts we are monitoring have been generated subsequent to functional knockdown of REF/Aly. The accumulation of our candidate transcripts was significantly lower when REF/Aly was depleted in these 4SU labeling assays (Figure 3B), mirroring the steady-state analyses (Figure 2B). Importantly, RNA was not recovered in the no-4SU control sample, demonstrating that we are assaying 4SU-containing RNAs rather than background signal. These data can be interpreted in two ways. First, REF/Aly depletion may decrease the synthesis rates of these transcripts. Second, REF/Aly may protect transcripts from a rapid decay process that functions within 2 h. In either case, these data show that REF/Aly effects on candidate transcripts occurs relatively early in their biogenesis.

To determine if changes in the abundance of candidate transcripts upon REF/Aly depletion is a result of decreased RNA stability, we performed transcription inhibition experiments using the transcription inhibitor Actinomycin D (ActD). Following a 96-h depletion of REF/Aly, cells were treated with 1 μg/ml ActD and RNA was harvested at time points from 0 to 4 h (0–240 min) after transcription shut-off. Comparison of the decay rates of specific RNAs in cells transfected with control siRNA or with REF/Aly siRNAs revealed no differences in RNA stability when REF/Aly was depleted (Figure 3C). We therefore conclude that changes in transcript abundance were not due to changes in RNA stability, at least over a 4-h period. Because the 4SU labeling experiments (Figure 3A and B) demonstrated that the effects of REF/Aly are observed within 2 h, these data support the conclusion that REF/Aly depletion decreases the transcription rates of the candidate mRNAs.

### REF/Aly depletion leads to diminished pol II occupancy on target genes

To more directly assess REF/Aly's role in transcription, we employed ChIP to look at polymerase density on our genes of interest. Following a 96-h knockdown, ChIP was performed with a pol II antibody that preferentially recognizes a hypophosphorylated form of the CTD (8WG16) (63). Five amplicons spanning the TLCD1 and THTPA loci were designed, as indicated in Figure 4A. Additionally, an amplicon located on chromosome 17 in a region with no annotated gene was used as a negative control. Pol II signal peaked in the amplicons closest to the transcription start site (TSS) for both TLCD1 and THTPA, as expected for recognition of the hypophosphorylated CTD (64) (amplicons 3 and 2, respectively, see black bars in Figure 4B). Interestingly, when REF/Aly was depleted, a significant decrease in pol II signal was observed for both TLCD1 and THTPA. These data demonstrate that depletion of REF/Aly results in a decrease in pol II density on two genes that were identified in the RNA-Seq as having decreased expression levels when REF/Aly is depleted. We could not examine changes in transcription for FXYD6 or loc10012881 because both of these genes overlapped with neighboring genes, precluding the design of unique ChIP amplicons. However, taken with the Act D and 4SU experiments (Figure 3), these data suggest that REF/Aly is important for transcription of at least two candidate genes, TLCD1 and THTPA.

Trimethylation of lysine 36 on histone 3 (H3K36me3) is a marker for active chromatin found preferentially at the 3′ ends of genes (65). H3K36me3 marks are linked to RNA processing factors and intron–exon boundaries (15,22,66). In addition, REF/Aly binds to Iws1, which is required for H3K36 trimethylation on at least some genes (46,67). Therefore, we reasoned that REF/Aly might be important for the H3K36 methylation of our candidate genes. To test this, we employed ChIP using an antibody that recognizes H3K36me3. As expected, H3K36me3 signal peaked in the 3′-most amplicon for both TLCD1 and THTPA (Figure 4C). However, when REF/Aly was depleted, no changes in H3K36me3 patterns were seen. Thus, even though REF/Aly depletion changes pol II density, it does not alter H3K36 trimethylation patterns on our candidate genes. Together, our data suggest that REF/Aly is necessary for efficient pol II loading, but its absence does not lead to a fully ‘closed’ chromatin state.

### Candidates are down-regulated soon after REF/Aly depletion

While REF/Aly depletion affects the steady-state levels of specific transcripts, we cannot conclusively state that these are due to direct effects of REF/Aly. For example, changes in RNA levels and transcription of our candidate transcripts could result from cell toxicity or other indirect effects. If so, we would expect direct effects of REF/Aly to be temporally linked to its depletion; that is, direct targets should be diminished relatively quickly upon REF/Aly depletion. To test this, we depleted REF/Aly for 48, 72 or 96 h and measured RNA levels by RT-qPCR. As seen in Figure 5A, by 48 h, candidate RNA levels are reduced by ∼50% even though residual amounts of REF/Aly were still present (Figure 1A) and no changes in growth rate (Figure 1A) or cell morphology (data not shown) were observed at this time. RNA levels continue to diminish over time correlating with REF/Aly depletion (Figure 5A). While this observation is by no means a proof of a direct effect of REF/Aly, it is consistent with the idea that changes in RNA levels are a direct result of REF/Aly depletion and not a result of toxicity.

### Candidate transcripts are preferentially bound by murine REF/Aly

Because REF/Aly is an RNA-binding protein, we hypothesized that REF/Aly binds directly to our candidate RNAs. To test this, we employed UV irradiation cross-linking followed by RNA immunoprecipitation (49). 293A-TOA cells were transfected with an expression construct for murine REF2-I protein that is N-terminally Flag-tagged (Fl-REF). Murine REF2-I is nearly identical to human REF/Aly across the conserved N-terminal, C-terminal and RNA-binding domains. The only variability is in the N- and C-variable regions, which are shorter in mouse than human (36). Twenty-four hours after transfection, cells were exposed to UV light to covalently crosslink protein with RNA. Following crosslinking, cells were lysed under stringent conditions and Fl-REF was immunoprecipitated using an anti-Flag antibody. Co-immunoprecipitated RNA was then detected by RT-qPCR. As seen in Figure 5B, all four candidate RNAs were immunoprecipitated more efficiently than a 7SK control RNA. We chose 7SK as a negative control because it is transcribed by RNA polymerase III, and is therefore not predicted to bind REF/Aly. Importantly, when cells were not exposed to UV irradiation, no immunoprecipitation of any RNAs was observed. This indicates that REF/Aly interaction with RNA occurs in cells and is not a result of protein-RNA re-assortment after cell lysis (68). Because UV irradiation only cross-links proteins that are in direct contact with RNA (54), the UV-dependent immunoprecipitation of candidate RNAs with Fl-REF indicates that REF/Aly directly binds to the candidate transcripts. Interestingly, ACTB also bound to REF/Aly above the 7SK background, but it was not identified as a DEG. This is consistent with the steady-state analysis suggesting that ACTB levels may be influenced by REF/Aly (Figure 2). Perhaps REF/Aly binds to a subfraction of ACTB mRNAs. Alternatively, REF/Aly may bind to ACTB, but is not absolutely required for its expression. Regardless, the observation that the DEGs showed a higher percent binding than ACTB or 7SK is consistent with a direct role for REF/Aly in their expression.

### REF/Aly effects on gene transcription share both general and specific properties

**Figure 6.**
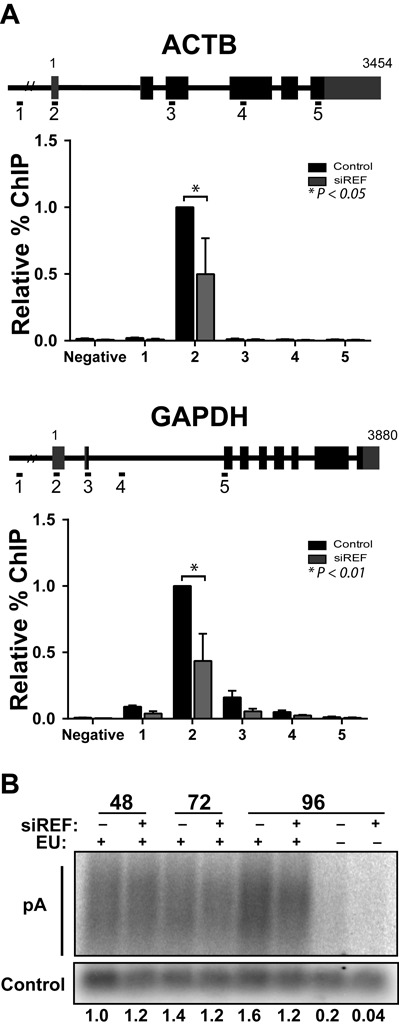
REF/Aly depletion decreases pol II occupancy on ACTB and GAPDH. (A) Pol II ChIP analysis of β-Actin (ACTB) or GAPDH loci after a 96-h REF/Aly knockdown. Percent immunoprecipitation is plotted relative to signal in the siControl sample closest to the TSS (ACTB #2, GAPDH #2). Error bars represent standard deviation from mean (*n* = 3). (B) Bulk poly(A) tail analysis of RNAs labeled for 2 h with EU following transfection of siControl or siREF for 48, 72 or 96 h. An exogenous biotinylated oligonucleotide was added to each sample to control for RNA recovery and gel loading. Poly(A) signal from northern blots were quantified (*n* = 2), normalized to the biotinylated oligonucleotide signal and signal relative to the 48-h control is indicated below each lane.

REF/Aly has been ascribed both general and gene-specific roles in RNA processing. Our analysis suggests that, while some transcript levels are more robustly influenced by REF/Aly depletion, the effects of REF/Aly may extend beyond the identified DEGs (Figures 2, 3B and 5). To see if the effects are at the transcription level, we employed pol II ChIP assays to examine the housekeeping genes β-Actin (ACTB) and glyceraldehyde 3-phosphate dehydrogenase (GAPDH), neither of which was identified as a DEG in all three samples in the RNA-Seq analysis. ChIP assays showed an ∼2-fold decrease in pol II density at the 5′ ends of both the ACTB and GAPDH genes (Figure 6A), suggesting that REF/Aly is important for transcription of additional genes not identified in the RNA-Seq. These data are consistent with the idea that REF/Aly acts as a general transcription factor or that cells are globally shutting down transcription in response to REF/Aly depletion. To assess global mRNA production, we depleted REF/Aly and examined the generation of newly made bulk poly(A) RNA using a labeling strategy similar to the 4SU experiments described above (57). Following 48, 72 or 96 h knockdown, cells were incubated for 2 h with an alkyne-modified uridine analog, 5-EU. Like 4SU, EU is incorporated into nascent RNA during the pulse and can be conjugated with biotin to capture the RNAs generated during the 2-h pulse (Figure 3A). Following streptavidin selection, RNA was treated with RNase T1, a G-specific endonuclease, to degrade all RNAs while leaving the poly(A) tails intact. Poly(A) RNA was then detected by northern blot. As seen in Figure 6B, bulk poly(A) RNA levels did not change as a result of REF/Aly knockdown, whether REF/Aly was depleted for 48, 72 or 96 h. We therefore conclude that depleting REF/Aly does not lead to a global decrease in mRNA transcription. However, the transcription of some genes that were not identified in the RNA-Seq analysis may also be REF/Aly-dependent. When taken together, our data suggest that REF/Aly contributes to the efficient transcription of a wide spectrum of genes, but it is not absolutely required for global transcription.

### Depletion of REF/Aly decreases the density of active pol II on candidate genes

**Figure 7.**
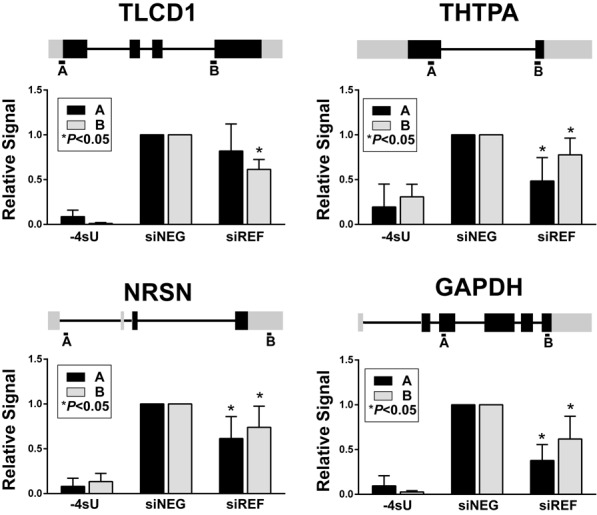
Depletion of REF/Aly alters active pol II density on candidate genes. Cells were treated with control siRNAs (siNEG) or REF/Aly siRNAs (siREF) for 72 h. NROs were performed with 4SUTP to capture nascent transcripts and RNAs were analyzed by RT-qPCR. Two amplicons (A and B) were tested per gene as shown on the gene diagrams above each panel. The signal was normalized to the siNEG for each experiment. The *P*-values are from a two-tailed, unpaired Student's *t*-test. In the ‘-4SU’ samples, UTP was substituted for 4SUTP in control siRNA-treated cell nuclei. These samples provide an important measure of the background to assess the signal-to-noise in this procedure, which varies considerably among genes.

Our ChIP assays demonstrated that REF/Aly depletion correlates with decreased pol II occupancy on candidate genes, but most of the detected pol II signal was at promoters (Figures 4 and 6). Moreover, our ChIP antibody preferably recognizes the hypophosphorylated form of pol II, not the elongation competent form (6,63). To determine if REF/Aly influenced the density of active pol II on candidate genes, we performed NRO assays in cells in which REF/Aly had been knocked down for 72 h. We employed a 4SUTP strategy to label nascent transcripts in isolated nuclei and subsequently recovered 4SU-containing RNA by biotinylation and streptavidin selection (59). We found that active pol II density on the candidate transcripts TLCD1 and THTPA as well as GAPDH decreased significantly after REF/Aly knockdown (Figure 7). To further demonstrate that REF/Aly is important for transcription of not only TLCD1 and THTPA, we tested an additional candidate gene identified in the RNA-Seq analysis, NRSN. Like TLCD1 and THTPA, NRSN1 transcription decreased when REF/Aly was depleted. Taken together, these data demonstrate that REF/Aly depletion leads to lower transcription rates of the candidate genes identified in our RNA-Seq analysis.

## DISCUSSION

Much work has been done demonstrating the importance of coupling between nuclear events in gene expression. The data presented here suggest that the RNA export factor, REF/Aly, may be an important component linking transcription, RNA processing and RNA export. While REF/Aly and its homologs function in mRNA export, REF/Aly is also capable of stabilizing a viral nuclear RNA and has been implicated in transcriptional control. Our RNA-Seq analysis suggests that a considerable number of transcripts are affected by REF/Aly upstream of export (Figure 1). We chose four candidates and confirmed that depletion of REF/Aly results in decreased steady-state levels (Figure 2). Moreover, stability analysis, metabolic labeling, ChIP and NRO assays demonstrate that the transcription of these candidates is impaired upon REF/Aly depletion (Figures 3 and 4). Additionally, REF/Aly binds directly to candidate transcripts (Figure 5), supporting a direct role for REF/Aly in their biogenesis. Together, these data suggest that REF/Aly promotes transcription of a subset of genes and may indicate that REF/Aly plays a functional role in linking splicing and export with transcription.

Our work shows that depletion of REF/Aly leads to decreased transcription of particular transcripts, but we cannot conclusively state whether that role is direct or indirect. REF/Aly plays important roles in the cell, particularly in facilitating export of mature mRNAs into the cytoplasm. In our studies, we have depleted REF/Aly, potentially disrupting important cellular processes and causing non-specific decreases in gene expression. Thus, we must allow for the possibility that changes in steady-state RNA levels, and particularly changes in transcription, are a result of indirect effects. However, several pieces of data argue against that idea. After a 48-h knockdown of REF/Aly, a significant decrease in candidate steady-state RNA levels was observed (Figure 5A), even though REF/Aly is only partially depleted at this time point. Importantly, no changes in cell viability or morphology were observed at this time (Figure 1A, data not shown), suggesting that changes in gene expression at these earlier time points are not caused by indirect effects, such as cell toxicity. Additionally, REF/Aly preferentially binds to candidate transcript RNAs when compared to a control RNA. Thus, REF/Aly binding to these transcripts may provide a direct link between REF/Aly, the bound mRNA and the transcription machinery, but more work is necessary to support this hypothesis.

So how might REF/Aly lead to increased transcription efficiency? The TREX complex, including REF/Aly, is deposited on a transcript coincident with splicing of the 5′-most intron, a process that occurs co-transcriptionally. Thus, when REF/Aly is bound to a nascent RNA, it is located in close proximity to the chromatin and transcription complexes. The close vicinity of REF/Aly to transcription complexes may provide it with an opportunity to influence transcription. In support of a direct role of REF/Aly in transcription, studies in both yeast and human cells have shown that REF/Aly (yeast homolog, Yra1) interacts with pol II. In yeast, Yra1 is both directly and indirectly associated with pol II. Specifically, work from Mackellar *et al.* has shown that *in vitro*, Yra1 is capable of binding directly to the hyperphosphorylated CTD (69). Additionally, Yra1 is recruited to the transcription sites through interaction with the 3′-end processing factor, Pcf11 as well as through association with THO (70,71). While no direct association of REF/Aly with the CTD has been demonstrated in metazoan cells, REF/Aly does indirectly interact with pol II through Spt6 and Iws1 in human cells (46).

Furthermore, Damgaard *et al.* established that mutation of the 5′ splice site reduces steady-state RNA levels by impairing transcription (14), and they show that the 5′ splice site enhances transcription by increasing recruitment of PICs to gene promoters. Interestingly, a 5′ splice site, even in the absence of splicing, is capable of stimulating transcription, so it seems that nascent transcripts can recruit RNA-binding factors, U1 snRNP in this case, where they jointly serve to process RNA and to stimulate transcription. It is possible that REF/Aly similarly promotes recruitment or activity of transcription factors after its binding to the nascent transcript. Moreover, work by Neugebauer *et al.* (16) demonstrated that ChIP peaks of the activating histone mark, H3K4me3, coincide with the 5′ splice sites of first introns. Both strong H3K4me3 peaks and H3K4me3 positioning at 5′ splices site depends on splicing. The authors also showed that splicing enhances pol II density. It is therefore tempting to speculate that splicing of the first intron, and the resulting deposition of REF/Aly in the TREX complex specifically to the 5′-most exon, is linked to this phenomenon. However, detailed mechanistic studies need to be performed to test this hypothesis.

In a non-mutually exclusive model, REF/Aly could affect transcription directly by interacting with transcription factors. Indeed, REF/Aly was first identified by its ability to interact with AML-1 and LEF-1 transcription factors and it has been reported to enhance b-ZIP protein interactions with DNA (44,45). More recently, work from Osinalde *et al.* identified REF/Aly as an E2F2-interacting protein, and a modulator of E2F2-responsive genes (72). In their microarray analysis REF/Aly was found to influence the expression of ∼400 genes. Comparison with our RNA-Seq analysis revealed 149 E2F2 targets among the 4859 identified nuclear DEGs fraction (Supplementary Table S2).

Depending on the species and cell type, REF/Aly has been proposed to act as a general mRNA export factor or as a gene-specific factor. We cannot make definitive conclusions regarding the generality of REF/Aly in transcription from our data. Our RNA-Seq data led to the identification of only a subset of genes, suggesting a specific effect, but it is important to point out that normalization schemes used for RNA-Seq experiments can be biased against factors that have general effects (73). Consistent with the idea that REF/Aly function is not restricted to the identified DEGs, the GAPDH gene showed diminished pol II occupancy (Figure 6A), NRO signal (Figure 7) and decreases in RNA levels (Figures 2, 3 and 5), even though it was not identified in our RNA-Seq analysis. However, we also show that bulk levels of newly synthesized polyadenylated mRNAs are not diminished after REF/Aly depletion, showing that bulk poly(A) RNA production occurs at similar global levels when REF/Aly is knocked down (Figure 6B). Whether or not REF/Aly acts on a specific subset of genes, or plays a more global role in transcription remains to be more definitively characterized.

REF/Aly has been implicated in promoting mRNA export, transcription and nuclear RNA stability. However, the contributions of REF/Aly to each of these activities to the cell remain unknown. Our studies specifically sought to identify REF/Aly's roles in RNA stability and/or transcription, so we cannot make any judgments regarding whether export is the primary role of REF/Aly. Interestingly, our RNA-Seq data showed that virtually all of the DEGs identified in the cytoplasmic fractions were down-regulated, strongly supporting a role for REF/Aly in nuclear mRNA export (Figure 1B). Consistent with this idea, a significant fraction of the DEGs in the nuclear fraction were up-regulated (Figure 1B), so it seemed reasonable that these transcripts accumulate in the nucleus upon export block. Surprisingly, however, among the transcripts that were down-regulated in the cytoplasm, only ∼10% were also identified as being up-regulated in the nucleus (Supplementary Table S1). While this suggests that the differential expression of mRNAs identified in the RNA-Seq is not due to export block, our analysis was not optimized to identify targets of REF/Aly mediated mRNA export and further bioinformatic analyses must be performed to directly test this idea. In addition, we were surprised that none of our candidates appeared to have altered stability upon REF/Aly knockdown (49–51). Of course, until we assay candidates in addition to the four examined here, we cannot rule out that other candidates were destabilized in the absence of REF/Aly. Therefore, our data do not demonstrate a role for REF/Aly in nuclear stability of endogenous transcripts, but this should not be interpreted to mean that no such activity is present.

Until recently, cross-talk between RNA processing events has been thought to take place in a forward manner. That is, transcription machinery positively influences factors involved in pre-mRNA processing. However, a growing body of evidence suggests that enhancement of gene expression occurs in a bidirectional fashion with RNA processing factors positively regulating transcription efficiency. Our work suggests that REF/Aly influences this bidirectional coupling but additional studies are required to determine the mechanisms of REF/Aly-mediated enhancement of pol II recruitment.

## SUPPLEMENTARY DATA

Supplementary Data are available at NAR Online.

SUPPLEMENTARY DATA
