# Supplementary material for: Depletion of REF/Aly alters gene expression and reduces RNA polymerase II occupancy
Source: Nucleic Acids Res. 2014 Dec 3;43(1):504–19. doi: 10.1093/nar/gku1278 (PMC4288173; doi:10.1093/nar/gku1278)
Supplement: SUPPLEMENTARY DATA [file supp_43_1_504__index.html]

Depletion of REF/Aly alters gene expression and reduces RNA polymerase II occupancy — Depletion of REF/Aly alters gene expression and reduces RNA polymerase II occupancy — Depletion of REF/Aly alters gene expression and reduces RNA polymerase II occupancy — SUPPLEMENTARY DATA 

# Depletion of REF/Aly alters gene expression and reduces RNA polymerase II occupancy

## SUPPLEMENTARY DATA

**Files in this Data Supplement:**

- SUPPLEMENTARY DATA
- SUPPLEMENTARY DATA
- SUPPLEMENTARY DATA
- SUPPLEMENTARY DATA
