# Supplementary material for: Depletion of REF/Aly alters gene expression and reduces RNA polymerase II occupancy
Source: Nucleic Acids Res. 2014 Dec 3;43(1):504–19. doi: 10.1093/nar/gku1278 (PMC4288173; doi:10.1093/nar/gku1278)
Supplement: SUPPLEMENTARY DATA [file supp_gku1278_nar-02107-v-2014-File009.pdf]

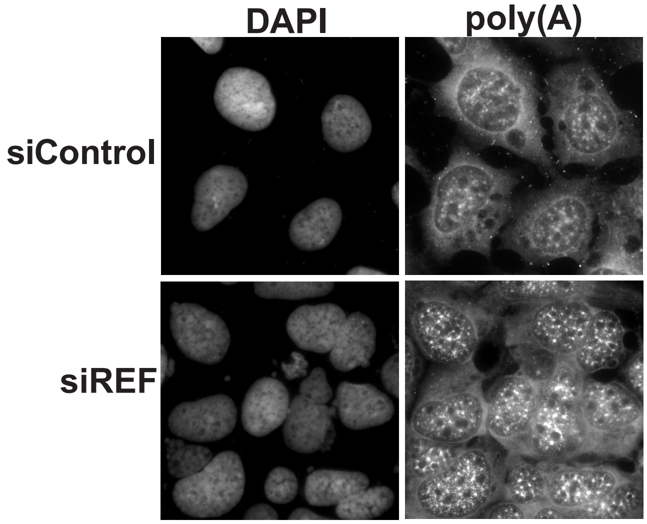

Supplemental Figure 1. Poly(A) RNA is localized to the nucleus and cytoplasm upon REF/Aly knockdown. FISH for poly(A)+ RNA following a 96-hr treatment with either control siRNA (siControl) or REF/Aly siRNAs (siREF).

Supplemental Figure 1

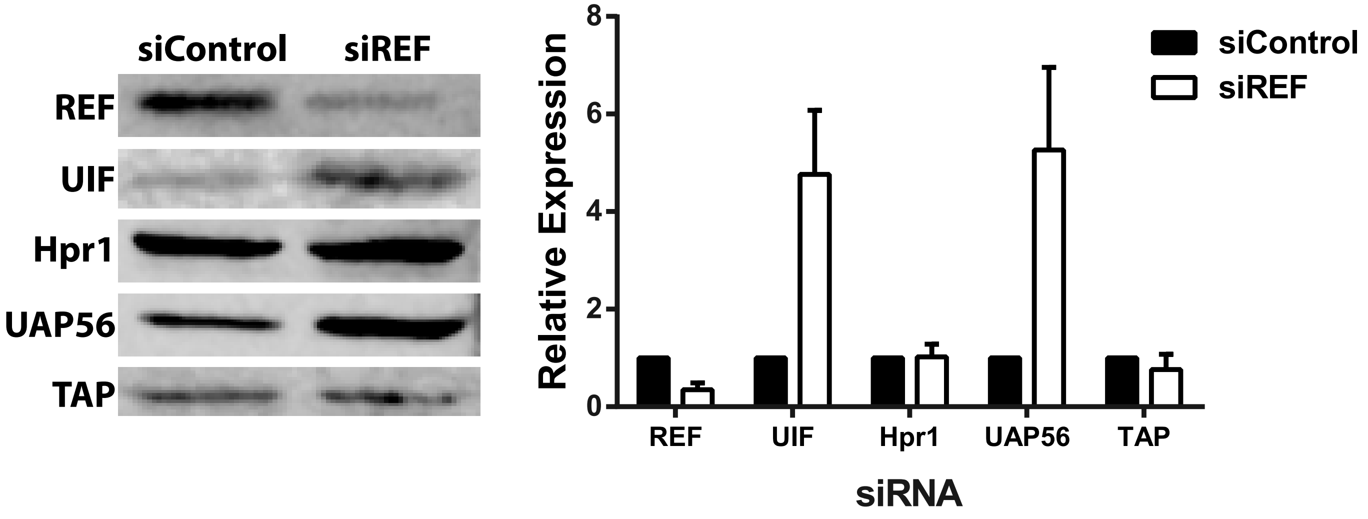

Supplemental Figure 2. Some TREX component levels are altered upon REF/Aly knockdown. Following a 96-hr depletion of REF/Aly, protein was harvested, separated by SDS-PAGE and immunoblotted for various TREX components. Representative western blots are shownon the left and quantification of protein levels is shown on the right (n=3). Protein expression is normalized to REF/Aly protein levels when cells were treated with siControl.

Supplemental Figure 2

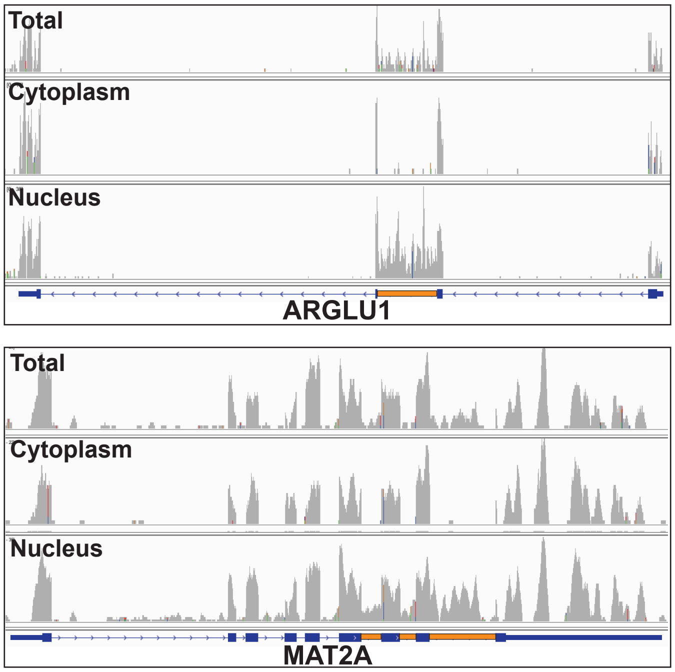

Supplemental Figure 3. Nuclear and Cytoplasmic fractionation is pure. Mapped reads of ARGLU1 and MAT2A from total, cytoplasmic, or nuclear samples. Areas colored in orange represent poorly spliced (retained) introns that accumulate in the nucleus. Transcripts containing introns are absent in the cytoplasmic fraction, demonstrating the purity of the fractionation process.

Supplemental Figure 3
